# Regulatory Effects of Statins on SIRT1 and Other Sirtuins in Cardiovascular Diseases

**DOI:** 10.3390/life12050760

**Published:** 2022-05-20

**Authors:** Danial Khayatan, Seyed Mehrad Razavi, Zahra Najafi Arab, Maryam Khanahmadi, Saeideh Momtaz, Alexandra E. Butler, Fabrizio Montecucco, Yuliya V. Markina, Amir Hossein Abdolghaffari, Amirhossein Sahebkar

**Affiliations:** 1 Department of Toxicology & Pharmacology, Faculty of Pharmacy, Tehran Medical Sciences, Islamic Azad University, Tehran, Iran; danialkhayatan@gmail.com (D.K.); mehradrazavi1376@gmail.com (S.M.R.); zahraa.naajafi@gmail.com (Z.N.A.); maryam.khanahmadi.1996@gmail.com (M.K.); 2 Medicinal Plants Research Center, Institute of Medicinal Plants, ACECR, Karaj, Iran; saeideh58_momtaz@yahoo.com; 3 Department of Toxicology and Pharmacology, School of Pharmacy, and Toxicology and Diseases Group, Pharmaceutical Sciences Research Center (PSRC), The Institute of Pharmaceutical Sciences (TIPS), Tehran University of Medical Sciences, Tehran, Iran; 4 GI Pharmacology Interest Group (GPIG), Universal Scientific Education and Research Network (USERN), Tehran, Iran; 5 Research Department, Royal College of Surgeons in Ireland, Adliya P.O. Box 15503, Bahrain; aeb91011@gmail.com; 6 First Clinic of Internal Medicine, Department of Internal Medicine, University of Genoa, 16132 Genoa, Italy; fabrizio.montecucco@unige.it; 7 IRCCS Ospedale Policlinico San Martino Genova-Italian Cardiovascular Network, 16132 Genoa, Italy; 8 Laboratory of Cellular and Molecular Pathology of Cardiovascular System, Avtsyn Research Institute of Human Morphology of FSBI “Petrovsky National Research Center of Surgery”, 3 Tsyurupy Str., 117418 Moscow, Russia; yu.v.markina@gmail.com; 9 Applied Biomedical Research Center, Mashhad University of Medical Sciences, Mashhad, Iran; 10 Biotechnology Research Center, Pharmaceutical Technology Institute, Mashhad University of Medical Sciences, Mashhad, Iran; 11 Department of Biotechnology, School of Pharmacy, Mashhad University of Medical Sciences, Mashhad, Iran

**Keywords:** sirtuins, statins, cardiovascular diseases, HMGCoA reductase inhibitors

## Abstract

Adverse cardiovascular disease (CVD) outcomes, such as sudden cardiac death, acute myocardial infarction, and stroke, are often catastrophic. Statins are frequently used to attenuate the risk of CVD-associated morbidity and mortality through their impact on lipids and they may also have anti-inflammatory and other plaque-stabilization effects via different signaling pathways. Different statins, including atorvastatin, rosuvastatin, pravastatin, pitavastatin, and simvastatin, are administered to manage circulatory lipid levels. In addition, statins are potent inhibitors of 3-hydroxy-3-methylglutaryl coenzyme A (HMGCoA) reductase via modulating sirtuins (SIRTs). During the last two decades, SIRTs have been investigated in mammals and categorized as a family of nicotinamide adenine dinucleotide (NAD)-dependent histone deacetylases (HDACs) with significant oxidative stress regulatory function in cells—a key factor in extending cell lifespan. Recent work has demonstrated that statins upregulate SIRT1 and SIRT2 and downregulate SIRT6 in both in vitro and in vivo experiments and clinical trials. As statins show modulatory properties, especially in CVDs, future investigations are needed to delineate the role of SIRT family members in disease and to expand knowledge about the effects of statins on SIRTs. Here, we review what is currently known about the impact of statins on SIRTs and how these changes correlate with disease, particularly CVDs.

## 1. Introduction

Cardiovascular diseases (CVDs) are the most common cause of death globally. Several factors, such as coronary atherosclerosis, lead to progression of CVDs. Complex interactions between inflammatory and metabolic processes lead to initiation and progression of coronary atherosclerosis. Genetic and mechanistic analyses have shown that lipoproteins, including apolipoprotein B (ApoB) and, in particular, low-density lipoprotein cholesterol (LDL-C), play a crucial role in atherogenesis. Improvement in CVD treatment, and especially acute myocardial infarction, has significantly increased average life expectancy. Given that about 20% of the world’s population will be aged 65 or older by 2030, an exponential increase in CVD prevalence is predicted, as many more people will have coronary heart disease, stroke, heart failure, and hypertension. In parallel, the prevalence of metabolic syndrome and diabetes is predicted to increase markedly in elderly individuals, further promoting CVD morbidity and mortality [1]. It is believed that beta-blockers, angiotensin-converting enzyme (ACE) inhibitors, and diuretics, which are currently used for the prevention of CVDs, prevent the onset and/or progression of CVDs as well as attenuate symptoms. Statins act to prevent CVDs and modulate circulating lipid concentration through a reduction in the biosynthesis of cholesterol, thereby leading to hepatic upregulation of low-density lipoprotein (LDL) receptors and enhanced LDL-cholesterol (LDL-C) removal from the bloodstream. Clinical trials have shown the efficacy of statins for both primary and secondary prevention of coronary heart disease. The effect of statins may be dependent or independent of LDL-C. In addition, clinical studies have demonstrated the advantages of statins in diseases that are not explicitly aligned with LDL-C, though some of these findings can be directly related to a reduction in cholesterol [2]. Statins are mainly administered as HMGCoA reductase inhibitors due to their strong efficacy in reduction of LDL through suppression of cholesterol synthesis. Statins are further utilized to reduce the risk of coronary heart disease due to their pleiotropic properties, such as elevating eNOS activity, restoring or improving endothelial function, suppressing platelet aggregation, decreasing oxidative stress damage and inflammation, and increasing the stability of atherosclerotic plaque through various signaling pathways [3,4,5,6,7,8,9,10,11,12]. A drug’s pleiotropic effects may or may not be associated with its primary mechanism of action. These and other emerging features might work in concert with statins’ LDL-C-lowering actions [2,13,14]. A more recently recognized pharmacological action of statins relates to its correlation with normalization of endothelial function, thus acting in the prevention of atherosclerosis at an early phase. Moreover, statins prevent dysfunction of smooth muscle cells and suppress the migration and activation of macrophages [15]. This suppression impacts the endothelium by preventing macrophage influx and may represent the mechanism by which signaling cascades lead to atherosclerosis and block the normal activity of endothelial cells, which statins restored. Given the pleiotropic effects of statins and their anti-inflammatory properties [16,17,18,19,20,21,22,23,24], statins are widely used in the treatment of atherosclerotic CVD. Further understanding of the mechanisms involved in the development of atherosclerosis may further guide the design of anti-dyslipidemia therapies. Much, however, is already known about the complex interactions that contribute to inflammation, which involve mRNA activity; the NOD-, LRR-, and pyrin domain-containing protein 3 (NLRP3) inflammasome; Krüppel-like factor 2 (KLF-2); peroxisome proliferator-activated receptor γ (PPAR-γ); Wnt inhibitor Dickkopf-1 (DKK-1); extracellular-signal-regulated kinase 5/nuclear factor erythroid 2-related factor 2 (ERK5/Nrf2); and sirtuin (SIRTs) pathways [25]. SIRTs, silent mating information-regulating analogs, are involved in many pivotal regulatory signaling pathways in cells that are associated with metabolism, stress response, aging, and the development of cancer and chronic disease. All members of the SIRT family exert actions that can be reduced or induced upon deacetylation [26]. SIRTs preserve the stability of DNA and cells against oxidative stress by targeting cellular proteins, such as PPAR-γ and its coactivator (PGC-1α), AMPK, forkhead transcriptional factors, NF-κB, p53, eNOS, and protein tyrosine phosphatase [27]. Here, the effect of statins on SIRT signaling pathways is reviewed in general, with a specific emphasis on their effects in CVDs.

## 2. Cholesterol, Inflammation and Cardiovascular Diseases (CVDs)

There are several factors associated with cardiovascular diseases (CVDs), including arterial hypertension, obesity, age, serum uric acid levels [28], and cholesterol. Although cholesterol is known to be associated with the development of CVDs, other factors (i.e., hypertension, obesity) have been implicated in promoting CVD [29]. For instance, it has been reported that uric acid is associated with kidney disease and cardiovascular conditions, such as hypertension and coronary artery disease [30]. Observational studies have shown that blood pressure levels are strongly correlated with the relative risk of stroke and heart disease. In line with the results of these studies, a systolic blood pressure of 140 mm Hg is considered to be optimal for preventing the adverse consequences of elevated blood pressure [31,32]. In addition, obesity has been recently demonstrated to be associated with cardiac structural changes independently of atherosclerotic diseases [29]. Nearly a century ago, a positive correlation between CVD and serum cholesterol was reported. Subsequently, several epidemiological and clinical investigations have established the association between increased circulating cholesterol and CVDs, especially atherosclerosis. In fact, the correlation between the level of cholesterol and death from coronary heart disease (CHD) is linear, with each 0.5 mmol/L (20 mg/dL) increase in total cholesterol leading to a 12% rise in mortality from CHD. Reduction in cholesterol levels leads to reduction in CVD mortality [33]. Statins reduce cholesterol biosynthesis in the mevalonate (MVA) pathway and modulate inflammation, as a pleiotropic effect, which helps to reduce the incidence of atherosclerotic cardiovascular disease (ASCVD), including cardiovascular death and all-cause mortality [34]. HMG-CoA is a biosynthetic intermediate for cholesterol and other isoprenoids, such as farnesyl pyrophosphate and geranylgeranyl pyrophosphate. Isoprenoids are important in cell proliferation and migration, as well as atherogenesis and vasculopathy-related inflammatory processes. A growing body of evidence suggests that statins have pleiotropic effects by inhibiting the generation of isoprenoid intermediates during cholesterol biosynthesis [2]. In general, lowering blood cholesterol levels, mainly LDL-C, attenuates vascular deposition and retention of cholesterol and apoB-containing lipoproteins, which are atherogenic [34]. Patients suffering from hyperlipidemia are almost twice as likely to develop CVD as those with normal concentrations of total cholesterol. Hence, early detection and management of hyperlipidemia is imperative for decreasing CVDs and preventing premature death [33]. Abnormal blood flow and plaque aggregations in the ventricle of the heart can provoke myocardial infarction, leading to congestive heart failure. Atherosclerosis is the most common coronary artery disorder, in which proliferation of fibrous tissue in the arterial wall occurs. Moreover, multiple factors, such as inflammation, with the related action of leukocytes, endothelium, and smooth muscle cells, along with low density lipoprotein (LDL) uptake, are critical factors in atherosclerosis progression and myocardial infarction. LDL does not infiltrate the endothelium of blood vessels in normal healthy conditions. However, LDL can pass through the endothelium to the sub-endothelium with subsequent formation of plaques, and abnormal endothelial cells are associated with LDL infiltration in this process. Furthermore, several signaling pathways have been associated with inflammation, including the NF-κB-, NLRP3-, PPAR-, and sirtuin-related pathways, all of which can be restored by appropriate therapies, such as statins [35].

## 3. Sirtuins and Related Signaling Pathways

**Figure 1:**
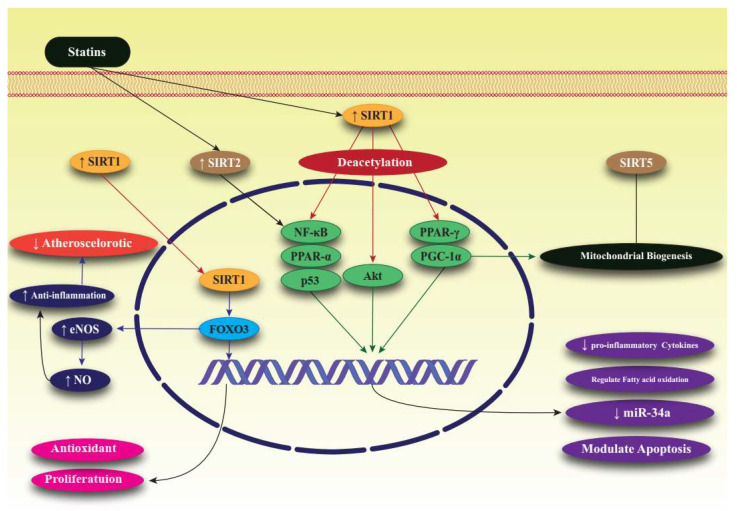
Effect of statins on SIRTs, particularly SIRT1, and related signaling pathways associated with lipid regulation. Forkhead box, class O3: FoxO3; nitric oxide: NO; endothelial nitric oxide: eNOS; peroxisome proliferator-activated receptor α: PPAR-α; peroxisome proliferator-activated receptor γ: PPAR-γ; nuclear factor kappa-light-chain-enhancer of activated B cells: NF-κB; sirtuin: SIRT; peroxisome proliferator-activated receptor gamma coactivator 1-alpha: PGC-1α; protein kinase B: Akt; microRNA-34: miR-34.

SIRTs are members of a large family of protein-modifying enzymes and NAD+-dependent deacetylators found in almost all organisms. The discovery of SIRTs as transcriptional silencing regulators of mating sites in yeasts attracted a great deal of attention [36]. The chemical structure of SIRTs is such that their enzymatic activities are regulated by various metabolites. Enzymatic reactions of SIRTs require NAD+ as a substrate, the concentration of which is determined by the nutritional status of the cell. SIRTs are completely dependent on NAD+, and the frequency of NAD+ and its breakdown in cells is closely related to the enzymatic activity of SIRTs. SIRTs convert NAD+ to nicotinamide which, in higher concentrations, can bind non-competitively and inhibit the activity of SIRTs [37,38]. SIRTs act in different parts of the cell as, for example, the acetylation of transcriptional regulators occurs in the nucleus and for other proteins occurs in the cytoplasm and mitochondria. These specific enzymes have important regulatory roles, such as regulating longevity in cells and organisms, fat motility in human cells, insulin secretion, cellular response to stress, enzyme activity, and basal transcription factor activity [39,40]. In mammals, the SIRT family consists of seven proteins that differ from each other in terms of enzymatic activity, tissue properties, and functions. Sirtuin has been studied in the context of prevention of diseases associated with aging and the maintenance of metabolic homeostasis. SIRT1, present in the nucleus and cytosol, appears to be the only intervention that promotes increasing life expectancy [41]. SIRT2 is a NAD+-dependent histone deacetylase that acts as an energy sensor and transcription effector by controlling histone acetylation. These enzymes not only acetylate histones, but also destroy a wide range of transcriptional regulators, thereby controlling their activities. SIRT2 is mainly considered to be a cytosolic enzyme, but is also present in the nucleus [42]. SIRT3, SIRT4 and SIRT5 have a mitochondrial targeting sequence, and SIRT6 and SIRT7 are nuclear enzymes. Further studies are underway to determine SIRT7′s exact site of activity [43]. SIRTs can play a key role in various pathologies because they stimulate the activity of mitochondria and mitochondrial proteins. SIRTs regulate fat and glucose metabolism in response to physiological changes and, therefore, act as vital network regulators that control energy homeostasis and determine life expectancy in cells and organisms. SIRT activation occurs not only in metabolic diseases, such as diabetes and obesity, but also in Alzheimer’s, Parkinson’s, and other neurodegenerative diseases and heart disease [44,45]. Though SIRTs are recognized as crucial targets for many diseases due to their wide and important physiological effects, the types of SIRTs and the pathways through which they exert their effects differ in different diseases. In mice, SIRT1 prevented diabetes, particularly in aged mice. The mediator NAD+ improved age-related type 2 diabetes in high-fat-fed mice through activation of SIRT1 [36,37,46,47]. SIRT1 was shown to increase insulin sensitivity by suppressing PTP1B tyrosine phosphatase and by increasing SIRT1 secretion through suppressing uncoupling protein 2 (UCP2). In addition to its positive effects in diabetes, SIRT1 in the hypothalamus positively affects the liver, muscle, and fat cells by, for example, stimulating adipogenesis, increasing insulin secretion, and by regulation of glucose homeostasis [48]. In relation to heart disease, increasing SIRT1 together with calorie restriction caused deacetylation and activation of eNOS, which ultimately increased NO, thereby dilating and protecting blood vessels [49]. SIRT2 can also redistribute endothelial cells in response to angiotensin II and mechanical traction by acetylating microtubules, and effects vascular regeneration in the setting of hypertension. SIRT3 can prevent cardiac hypertrophy by modulating mitochondrial homeostasis, and overexpression of SIRT6 suppressed angiotensin II-induced cardiomyocyte hypertrophy [50,51,52]. SIRT1 improves learning and memory in mice, and its expression in the hippocampus caused effects on ERK1/2 phosphorylation and changes in the expression of genes involved in synaptic function [53]. In Alzheimer’s disease, SIRT1 prevented axonal degeneration and neurodegeneration, and also reduced tau proteins by deacetylating tau and reducing the production of beta-amyloids [54]. In animal models of Huntington’s disease, high expression of SIRT1 improved the neuropathology and increased BDNF expression, as well as extending lifespan [55]. In Parkinson’s disease, expression of SIRT1 increased life expectancy and protected neurons against neurotoxicity [30]. Unlike SIRT1, which has protective effects in neurodegenerative diseases, SIRT2 is toxic to neurons and causes increased accumulation of beta-amyloids and other proteins, making cells more vulnerable to apoptosis [56,57]. SIRT1 has been shown to modulate cellular stress and survival via promoting tumorigenesis in various cancers, including breast, prostate, colon, and pancreas. However, SIRT1 could be a tumor suppressor. For instance, an in vivo study on SIRT1 mutant mice has shown genomic instability, impairment of DNA repair response, and elevated incidence of tumorigenesis. In addition, SIRT1 concentrations are lower in hepatic cell carcinoma and breast cancer. SIRT3 has also been suggested as a mitochondrial tumor suppressor, but overall, the main role of SIRT1 and SIRT3 in tumor suppression is controversial [58,59]. The protective role of SIRT2 against cancer has been observed in various studies [60,61]. SIRT2 can prevent the formation of colonies and suppress the growth of tumor cells in glioma cell lines [62]. SIRT6, a tumor suppressor, can also acetylate the H3K9 and H3K56 histones and plays a considerable role in DNA repair in two-strand breaks, but its overexpression in a variety of cancer cells leads to increased apoptosis [63]. Lipid metabolism involves the synthesis, uptake, storage, and utilization of lipids and requires careful control. SIRTs affect various aspects of fat homeostasis. When the body’s total energy storage is maximized, glucose, fatty acids, and excess amino acids are utilized in the liver to synthesize fatty acids, which are sent into the white adipose tissue and stored as TGs. Fatty acid synthesis occurs in the cytosol, and a key transcription factor, LXR, controls the expression of genes involved in lipid synthesis [64,65]. SIRT1 can degrade LXR and increase its transcriptional activity, ultimately enhancing fatty acid synthesis. SIRT1 can also inhibit the fluctuation (decrease or increase) of fat movement through lipolysis by suppressing PPAR-γ activity, which is the main regulator of fat cell differentiation [66,67]. SIRT2 may also inhibit lipid production and promote lipolysis by deacetylation. SIRT6 is also involved in controlling the synthesis of fatty acids [68,69]. Figure 1 summarizes the effect of statins on sirtuins, as well as their signaling pathways (Figure 1).

## 4. Evidence for Statins in the Regulation of Sirtuin-Mediated Activities

Statins are 3-hydroxy-3-methylglutaryl coenzyme A (HMGCoA) reductase inhibitors, which were first discovered in fungi. Despite the introduction of different classes of lipid-lowering drugs in the recent decades [70,71,72], statins have remained as the most commonly used drugs for the treatment of dyslipidemia, hypertriglyceridemia, and hypercholesterolemia; atorvastatin, simvastatin, fluvastatin, pravastatin, and rosuvastatin are examples. Statins can also be used therapeutically in diseases such as stroke, heart attacks, inflammatory diseases and cancer. These drugs vary in terms of efficacy, potency and side effects. Myopathy, rhabdomyolysis, headache, dizziness, and gastrointestinal (GI) dysfunction are common side effects of statins [73]. Proprotein convertase subtilisin/kexin type 9 (PCSK9) is a biomarker that has recently been considered as useful in CVDs. PCSK9 is a substantial player in hypercholesterolemia and may potentially play a function in atherosclerosis-related inflammatory pathways [74,75]. However, statins have been shown to have therapeutic effects in CVDs via regulation of inflammatory pathways such as eNOS and SIRTs. Administration of statins reduces SIRT signaling pathway expression, leading to an improvement in CVDs [76]. Table 1, Table 2 and Table 3 presents studies that have investigated the effects of statins on SIRT signaling pathways (Table 1, Table 2 and Table 3). It is possible that ezetimibe and bempedoic acid might also have impacts upon inflammatory mechanisms associated with dyslipidemia [77].

### 4.1. Statins Modulate Sirtuins in Inflammatory Conditions

In a 2020 study by Hong et al., atorvastatin (10 mg/kg per day) exhibited beneficial effects on bone mass and improved bone microarchitecture when used chronically for 12 weeks in a mouse model. Although atorvastatin decreased osteocalcin (OCN) in serum and inversely increased the OCN in bone tissue, the expression levels of ALP, SIRT1, and Runx2 were elevated. Furthermore, atorvastatin was shown to improve bone turnover balance and increase trabecular bone volume and bone formation in aged mice [78]. Available evidence also suggests that atorvastatin has favorable effects on neuronal cells. In 2019, Celik et al. reported that atorvastatin (1 μM) increased SIRT1 in human neuroblastoma cells, which could have protective effects in neuronal cells, although this agent also reduced the Sestrin2 that was induced by Aβ1-42 and inversely increased the expression of LC3II in neuronal cells [83]. In a 2018 study, Kim et al. showed that fluvastatin could act as a SIRT6 activator. Fluvastatin (5 μM) increased SIRT6 levels and increased its nuclear translocation in HEPG2 cells. Fluvastatin exerted its beneficial effects on SIRT6 via induction of AMPK phosphorylation. Moreover, the expression on LKB and SREBP1 phosphorylation was increased by this statin [94]. In a study using C57BL/6J mice, atorvastatin exhibited favorable effects on cognitive impairment via the SIRT1 signaling pathway in low and moderate doses, while there was no significant effect on SIRT1 when administering high doses; both inhibition and downregulation of SIRT1 were more pronounced when administering moderate doses. Furthermore, atorvastatin was shown to decrease the expression of interleukin-1β (IL-1β), tumor necrosis factor-α (TNF-α), and IL-6, and inversely increased the activity of superoxide dismutase (SOD) and catalase (CAT) [87]. Simvastatin was shown to improve mouse brain damage via the SIRT1 signaling pathway. In this study, C57BL/6J mice were pretreated with simvastatin (20 mg/kg) on three different days in a seven-day cycle. Simvastatin intensified the expression of Bcl-2 and PARP and reduced p53/p-p53 [88]. In 2013, Kok et al. investigated the effects of simvastatin in collagen-induced arthritis (CAI) and reported that simvastatin downregulated TNF-α expression and CCL20 production. Although it induced regulation of the SIRT1/FoxO3a signaling pathway, simvastatin showed therapeutic effects in CAI [85]. Additional studies indicated that simvastatin exerted its effect via the SIRT signaling pathways. Simvastatin (10 mg/kg) was found to exert its effect via SIRT1 in the rat model of acute pulmonary embolism (APE). The compound attenuated pulmonary inflammation and reduced the expression of inflammatory cytokines such as TNF-α, IL-1β, IL-6, and IL-8. Administration of simvastatin increased the expression of eNOS and SIRT2, and inhibited NF-kB expression [91]. Different doses of statins (10, 30 mg/kg) were reported to reduce SIRT6 expression via induction of miR-495 expression. An increase in hepatic gluconeogenesis was demonstrated in C57BL/6 male mice after administration of statins for three days [92]. De las Heras et al. reported that rosuvastatin increased insulin sensitivity in rat models via the SIRT1 signaling pathway. Rosuvastatin (15 mg/kg/day) mediated factors known to be involved in reducing insulin sensitivity in obese rats, such as TNF-α, CRP, VLDL, TG, cholesterol, and leptin, while increasing IL-6. Reduced levels of SIRT1, PPAR-γ, and GLUT-4 expression were normalized after administration of rosuvastatin for seven weeks [89].

### 4.2. Statins Modulates Sirtuins in CVDs

In 2014, Gong et al. reported that prolonged use of atorvastatin (5 mg/kg/day) could reduce endothelial cell damage. In this study, Wistar rats were treated with atorvastatin for eight months. The compound reduced malondialdehyde (MDA) and, conversely, increased SOD, eNOS, and SIRT1 expression. SIRT1 expression is related to the eNOS/iNOS ratio and improved age-dependent endothelial cell damage [90]. Moreover, in a study on EAhy 926 endothelial cells, where the effects of two statins (atorvastatin and pravastatin) on NOS, ROS, and SIRT levels were investigated, both statins were shown to increase SIRT1 and AMPK levels, with the phosphorylation and activation of SIRT1 being markedly elevated by pravastatin. Neither of the statins had any significant effects on histone H3 acetylation [80]. In 2010, Ota et al. reported an in vivo experiment demonstrating the anti-aging effects of statins on endothelial cells. In this study, atorvastatin, pravastatin, and pitavastatin were used in 50 and 100 nmol/L doses. SIRT1 and eNOS were found to be significantly increased due to Akt phosphorylation. Overall, the statins caused a reduction of senescence in endothelial cells [81]. In another in vivo study, atorvastatin (0.02–0.5 μM) and rosuvastatin (0.4–10 μM) were used to elucidate their effects on endothelial progenitor cells in CAD. Both atorvastatin and rosuvastatin increased SIRT1 levels, being most effective at 0.5 μM and 10 μM doses [82]. Lei et al. reported that simvastatin provided some anti-aging effects via the SIRT signaling pathway. Simvastatin (5 mg/kg/day) decreased lipoproteins, such as OX-LDL, LDL, and cholesterol, while enhancing SIRT1 expression. Therefore, increased SIRT1 expression could inhibit OX-LDL, which then ameliorated vascular endothelial cell damage [95]. In 2014, Gang et al. demonstrated that simvastatin (0.1, 0.01 μM) had beneficial effects on endothelial progenitor cells—administration of simvastatin elevated SIRT1 expression, especially at higher doses, and increased cell proliferation that was decreased by TNF-α administration. Moreover, simvastatin was shown to reduce apoptosis [86]. In STZ-induced diabetic mice, pitavastatin (3 mg/kg) improved mitochondrial biogenesis by regulating the SIRT1 signaling pathway. Pitavastatin (100 nmol/L) was sufficient to protect endothelial senescence and pitavastatin also increased the expression of eNOS and SIRT1 [81]. In an analytical study, administration of rosuvastatin (1, 10, 100 μM) enhanced SIRTs in endothelial cells (EAhy 926). Rosuvastatin had a higher induction effect on SIRT1 than SIRT2 [84]. In a clinical study conducted on 108 patients with a history of premature myocardial infarction, administration of atorvastatin and simvastatin was shown to reduce LDL levels, as expected, and increase SIRT1 expression over the three-month treatment period. Furthermore, atorvastatin and simvastatin decreased eNOS but had no significant effect on TOS, TAS, or OSI [93]. In another clinical study, similar results were obtained in 111 CAD patients, where atorvastatin and rosuvastatin were shown to enhance SIRT1 expression and decrease the levels of eNOS [3]. In a clinical study, seventy CAD patients received atorvastatin (10 mg) and rosuvastatin (2.5 mg), which led to a reduction in miR-34a levels in the atorvastatin group but not in the rosuvastatin group. Administration of atorvastatin increased the expression of SIRT1, but no change was observed in the rosuvastatin group [82]. SIRT3 deficiency causes cellular dysfunction and is related to the development of various diseases, including metabolic disorders, aging, and CVD [96]. The sirtuin members also may have negative effects on CVDs. In 2020, Wang et al. reported that in angiotensin 2 (ANG2)-treated mice, SIRT4 causes heart failure, fibrosis, and hypotrophy. SIRT4 stimulated mitochondrial fusion, inhibited mitophagy via association with the optic atrophy 1 (OPA1) protein, and increased ROS accumulation by interacting with SIRT3, blocking SOD2 activation in SIRT4-transfected HEK293 cells and fibroblasts [97]. By increasing oxidative stress, SIRT4 has been demonstrated to cause pathological cardiac hypertrophy in response to pressure overload. SIRT4 appears to block SIRT3-dependent activation of the manganese-dependent superoxide dismutase (MnSOD), an antioxidative enzyme [98]. In 2008, Lynn et al. showed SIRT2 has a detrimental impact on CVDs, hence its downregulation in H9c2 cells protects against ischemia-reperfusion (I/R) injury. Anoxia-reoxygenation injury has been demonstrated to upregulate SIRT2. Downregulation of SIRT2, on the other hand, upregulates 14-3-3 zeta and alters the subcellular location of the Bcl-2-associated death promoter from mitochondria to cytosol, resulting in a cardioprotective phenotype [99]. SIRT1 overexpression at high levels (more than 12 times above normal) has negative effects on cardiac function, but low to moderate overexpression of SIRT1 in transgenic mice prevents the age-dependent development of cardiac hypertrophy and fibrosis [100].

## 5. Conclusions and Perspectives

CVDs are a leading cause of morbidity and mortality. Various factors, such as environmental factors and lifestyle, play a significant role in the initiation and progression of CVDs. Patients are commonly prescribed multiple drugs from different classes, such as ACE inhibitors, statins, beta blockers, and calcium channel blockers, to exert their effects through specific mechanisms, such as modulating lipid levels, blood pressure, vessel diameter, blood flow volume, and the blockage or stimulation of particular receptors. The anti-inflammatory and lipid regulatory properties of statins are effected through different signaling pathways, particularly SIRTs. Each member of the SIRT family shows promising potential to modulate lipids such as LDL, LDL-C, and TG. SIRTs perform pivotal roles in balancing cellular homeostasis to preserve the homeostasis of cells, making them appropriate candidates for investigation as regulators of lipids. SIRT1 was found to be the most effective SIRT for the regulation of lipids. Additionally, other well-recognized risk factors, such as diabetes, obesity, CVDs involving CAD, acute pulmonary embolism, and atherosclerosis, can be attenuated by restoring the activity of SIRTs, especially SIRT1. Statins have been shown to reduce lipid levels via SIRT pathways (Figure 1). Upregulation of SIRT2 and downregulation of SIRT6 effectively regulates lipids, as documented in in vivo, in vitro, and clinical studies. Atorvastatin can also regulate lipids through its action on SIRT1. Hence, in-depth investigations are necessary to elucidate the precise functions of individual SIRTs and to determine whether the SIRTs have practical regulatory, overlapping functions that could prove crucial in regulating lipids in cells to prevent or mitigate pathological manifestations. More in vitro, in vivo, and clinical investigations would provide more information about the exact effects of each drug in the statin class and their effects on reduction of CVD symptoms through the SIRT-related signaling pathways. Overall, the potential of SIRTs as drug targets, and their effects on lipid regulation, could guide therapeutic approaches to treat abnormal concentrations of LDL, LDL-C, VLDL, and TG.

## Figures and Tables

**Table 1:** Effect of statins on SIRTs: in vitro studies.

Study Design	Disease	Intervention		Number of Cells		Treatment Duration	Results	Ref.
		Case	Control	Case	Control			
BMSCs of male apoE^–^^/^^–^	apoE^–^^/^^–^ deficient	Atorvastatin (0 10, 100, 1000 nM)	L-DMEM containing 10% fbs.	1 × 10^5^ cells	1 × 10^5^ cells	72 h	(1) ↑ expression of SIRT1, Runx2, ALP, and OCN (2) ↑ osteogenesis through activation of SIRT 1	[78]
Human THP-1 monocytic leukemia cells	Leukemia	Rosuvastatin (10 µM)	BSA/AGEs	Not mentioned	Not mentioned	24 h	(1) AGE-RAGE → ↓ SIRT1 gene expression through ROS production (2) Inhibition of AGE-induced superoxide production → restores ↓ SIRT1 mRNA levels.	[79]
EA.hy926 ECs	-	Atorvastatin (5 μM) Pravastatin (5 μM)	Untreated	Not mentioned	Not mentioned	48 h	(1) ↑ SIRT1 and SIRT3 (2) ↑ fraction of SIRT1 phosphorylation by pravastatin (3) No effect on histone H3 acetylation (4) ↓ availability of nicotinamide for NAD + synthesis (possibly limiting SIRT1 activity)	[80]
HUVEC	Senescence induced by H_2_O_2_	Atorvastatin (50 and 100 nM), Pitavastatin (50 and 100 nM), Pravastatin (50 and 100 nM)	Vehicle (0.05% DMSO)	1 × 10^5^ cells	1 × 10^5^ cells	24 h	(1) ↑ eNOS and SIRT1 expression (2) 100 nM of statin → protective effects against endothelial senescence	[81]
EPCs	CAD	Atorvastatin, (0.02–0.5 μM); Rosuvastatin, (0.4–10 μM) dissolved in DMSO	vehicle	8 × 10^6^ cells	8 × 10^6^ cells		(1) ↓ miR-34a levels and ↑ SIRT1 (changes were dose dependent and were more pronounced with atorvastatin)	[82]
SH-SY5Y	AD	Atorvastatin (1 µM) + Aβ1–42 (2.5, 5, 10 µM)	0.1% DMSO	Not mentioned	Not mentioned	40 h	(1) Reverse ↓ expression of SIRT1, induced by Aβ1–42 (As neuroprotective effect)	[83]
EA.hy926 ECs	CVD and diabetes	Rosuvastatin (1, 10 and 100 µM)	-	Not mentioned	Not mentioned	24 h	(1) ↑ SIRT1 expression (2) No change in SIRT2 expression	[84]
RASFs	RA	Simvastatin dissolved in ethanol (4 mg/mL)	Untreated	Not mentioned	Not mentioned	2 h	(1) ↑ SIRT-1 and SIRT-1/FoxO3a signaling → ↓ TNF-α-induced CYR-61and phospho-FoxO3a expression	[85]
EPCs	Atherosclerosis	Simvastatin (10, 100 nM)	-	4000 cells	4000 cells	72 h	(1) ↑ TNF-α-induced ↓ SIRT1 levels → inhibition cell apoptosis	[86]

**Table 2:** Effect of statins on SIRTs: in vivo studies.

Study Design	Disease	Intervention		Number of Animals		Treatment Duration	Results	Ref.
		Case	Control	Case	Control			
Male C57BL/6J mice	apoE^–^^/^^–^ deficient	Atorvastatin (10 mg/kg day, i.p.) dissolved in DMSO	DMSO (equivalent amount)	n = 12	n = 12	12 weeks	(1) ↑ trabecular bone volume and bone formation (2) ↑ SIRT1 expression in the bone tissue (3) Improvement in the balance of bone turnover	[78]
Male C57BL/6J mice	HFD-induced obesity	Atorvastatin (3, 6 or 12 mg/kg/day, p.o.)	Normal diet (3.5% fat)	n = 10	n = 10	7 months	(1) Activation of SIRT1 inhibition at moderate and low doses (2) High dose had no effect on SIRT1 activation. (3) Neuroprotective effect through activation of SIRT1 effect	[87]
Male C57BL/6J mice	Ionizing radiation-induced thymus damage	Pre-administrated simvastatin (20 mg/kg/day, i.g.), 1, 3 and 7 days, following 4 Gy ⁶⁰Co γ-radiation	0.5% CMC Na	n = 10	n = 10	14 days	(1) ↑ expression of Bcl-2 and PARP and ↓ p53/p-p53 (2) ↑ expression of AKT/SIRT1	[88]
Male WR	HFD-induced obesity	HFD+ rosuvastatin (15 mg/kg/day)	Normal diet (3.5% fat)	n = 10	n = 10	7 weeks	(1) Normalizing ↓ expression of SIRT-1, PGC-1α, PPAR-γ, and GLUT-4	[89]
Male C57/BL6 mice	STZ-induced diabetes	Pitavastatin (3 mg/kg /day, p.o.)	Vehicle	n = 7	n = 7	Lifetime of mice	(1) ↑ SIRT1 via the Akt pathway (2) Protective effect by interaction of SIRT1 with eNOS against endothelial aging (3) ↑ mitochondrial biogenesis by SIRT1-dependent manner → ↓ oxidative stress	[81]
Male WR	Vascular aging	Atorvastatin (5 mg/kg/day)	-	n = 8	n = 8	8 months	(1) ↑ SOD, NO, and eNOS expression. (2) ↑ expression of SIRT1 in ECs and VSMCs (3) SIRT1 is positively correlated with eNOS or eNOS/iNOS ratio and negatively correlated with iNOS.	[90]
Male SD rat	CIA	Simvastatin (0.5 mg/mL, i.a.), every 5 days	Normal saline, i.a. every 5 days	n = 20 (right ankle joint of 20 rat)	n = 20 (left ankle joint of 20 rat)	21 days	(1) ↓ CYR-61 expression → Improve arthritis	[85]
Male SD rat	APE	Simvastatin (10 mg/kg/day, i.g.)	Untreated	n = 24	n = 24	14 days	(1) ↑ mPAP, RVSP, and A-aDO2 and ↑ PaO2 (2) ↓ expression of TNF-α, IL-1β, IL-6, and IL-8 (3) ↑ mRNA expression of eNOS and SIRT2 (4) ↓ mRNA expression of NF-κB	[91]
Male C57/BL6 mice	Type 2 diabetes	Statins (10 or 30 mg/kg/day, i.p.) in DMSO in saline	Vehicle	Not mentioned	Not mentioned	3 days	(1) ↑ miR-495 expression → ↓ SIRT6 (2) No change in other SIRTs (3) ↑ mRNA levels of gluconeogenesis genes (4) Dysregulation of miR-495/SIRT→ FoxO1 upregulation	[92]

**Table 3:** Effect of statins on SIRTs: clinical studies.

Study Design	Disease	Intervention		Number of Patients		Treatment Duration	Results	Ref.
		Case	Control	Case	Control			
Retrospective study	STEMI	Simvastatin/Atorvastatin	Untreated	n = 79	n = 91	More than 3 years	(1) ↓ LDL (2) ↑ SIRT1 and ↓ eNOS levels (3) No changes in TOS, TAS, and OSI levels	[93]
Retrospective study	CAD	Atorvastatin and Rosuvastatin	untreated	n = 111 (n = 91 atorvastatin, n = 20 rosuvastatin)	n = 128	Not mentioned	(1) ↓ SIRT1 levels and ↑ eNOS levels (2) ↑ TAC, TOS levels	[3]
Randomized controlled study	CAD	Atorvastatin (10 mg/day, n = 35) or Rosuvastatin (2.5 mg/day, n = 35)	Non-CAD group (receiving statin)	n = 70	n = 48	8 months	(1) ↓ LDL-C and TAG levels (2) ↓ miR-34a and ↑ SIRT1 in the atorvastatin group and unchanged in the rosuvastatin group	[82]

Abbreviations of Tables: (BSA) bovine serum albumin; (AGEs) advanced glycation end products; (RAGE) receptor of AGEs; (CAD) coronary artery disease; (PCI) percutaneous coronary intervention; (HFD) high-fat diet; (HUVEC) human umbilical vein endothelial cells; (STZ) streptozotocin; (VSMCs) vascular smooth muscle cells; (OX-LDL) oxidized low-density lipoprotein; (SH-SY5Y) human neuroblastoma cells; (AD) Alzheimer’s disease; (eNOS) endothelial nitric oxide; (HUVEC) human umbilical vein endothelial cells; (ECs) endothelia cells; (EPCs) endothelial progenitor cells; (SOD) superoxide dismutase; (HepG2) hepatocarcinoma cell line; (HFD)high-fat diet; (apoE^–/–^) apolipoprotein E-deficient; (ALP) alkaline phosphatase; (OCN) osteocalcin; (RASFs) rheumatoid arthritis synovial fibroblasts; (RA) rheumatoid arthritis; (TAG) triacylglycerol; forkhead box protein O1; (STEMI) ST-elevation myocardial infarction; (TAS) total antioxidant status; (TOS) total oxidant status; (OSI) oxidative stress index; (AMPK) AMP-activated protein kinase; (SREBP)-1sterol regulatory element-binding protein; (APE) acute pulmonary embolism; (CIA) collagen-induced arthritis; (PPAR-γ) peroxisome proliferator-activated receptor-γ; (GLUT-4) glucose transporter type 4; (NAD+) nicotinamide adenine dinucleotide; (SIRT) sirtuin; (BMSCs) bone-marrow-derived mesenchymal stem cells; (qRT-PCR) quantitative real-time reverse transcription-polymerase chain reaction; (Runx2) runt-related transcription factor; (DMSO) dimethyl sulfoxide; (DMEM) Dulbecco’s Modified Eagle Medium; (p.o.) oral administration; (i.g.) intragastric; (i.a.) intraarticular; (i.p.) intraperitoneally; (WR) Wistar rats; (SD) Sprague–Dawley.

## Data Availability

Not applicable.
